# Hand-grip strength among older adults in Singapore: a comparison with international norms and associative factors

**DOI:** 10.1186/s12877-017-0565-6

**Published:** 2017-08-04

**Authors:** Hui Lin Ong, Edimansyah Abdin, Boon Yiang Chua, Yunjue Zhang, Esmond Seow, Janhavi Ajit Vaingankar, Siow Ann Chong, Mythily Subramaniam

**Affiliations:** Research Division, Institute of Mental Health, Buangkok Green Medical Park, 10 Buangkok View, Singapore, 539747 Singapore

**Keywords:** Anthropometry, Aging, Hand strength, Normative values, Older Adults, Singapore

## Abstract

**Background:**

Hand-grip strength (HGS) serves as a proxy measure for muscle function and physical health. Studies have shown that low HGS is associated with common age-related disorders including frailty and sarcopenia. The aim of the present study was to establish the normative values of HGS among older adults in Singapore and to compare it with data from Western and other Asian countries. The study also aimed to explore the sociodemographic and anthropometric correlates of HGS.

**Methods:**

Data were collected from 2043 men and women aged 60 years and above who took part in the Well-being of the Singapore Elderly study in 2013. HGS was obtained using a Jamar Plus + digital hand dynamometer. Normative data were stratified by; 5-year age groups, sex and ethnicity. Relationships between the HGS with various sociodemographic and anthropometric correlates were examined using multiple linear regression analysis.

**Results:**

The mean HGS demonstrate a decreasing trend with increased age across all ethnic groups and sexes. HGS among Singapore older adults were relatively low compared to Western and other Asian countries. Males in the youngest age group (60-64) and of Chinese ethnicity attained greater HGS values than their counterparts. When the regression analysis was stratified for sex, significant associations were found between height, upper arm circumference with HGS in the males sample, and between height, weight, waist circumference and HGS in the females sample.

**Conclusions:**

Older adults in Singapore have a relatively weak HGS compared to other countries. Greater height and weight, and smaller waist circumference are independently associated with greater HGS in females but not males. These results facilitate the interpretation of HGS conducting using Jamar digital-type dynamometers among the older adults in Singapore.

## Background

Hand-grip strength (HGS) plays an important role in the daily lives of people and serves as a reliable proxy indicator of an individual’s hand motor abilities. Many daily functions such as carrying require the use of the flexor musculature of the forearms and hands, and these are the muscles that are involved in gripping strength. Recent studies have reported the importance of HGS as it is used to help identify common age-related disorders such as frailty and sarcopenia [1, 2]. HGS can be measured using different assessors such as the Nintendo Wii Balance Board and Grip-ball [3, 4] or using different brands of dynamometer i.e. Rolyan, Smedley, and Jamar dynamometers [5, 6]. Hand-grip dynamometer was found to be a valid tool in clinical and research practice, and is an easy, quick, and inexpensive way of assessing HGS in older adults [3].

Several publications have appeared in recent years documenting HGS normative values in older adults in United States (US) [7], United Kingdom (UK) [8], Japan [9], Hong Kong [10], Taiwan [11, 12], Malaysia [13] and Singapore [6]. Dodds et al. [14] studied differences in HGS by world region and reported that the HGS normative data between the British and developed regions (e.g. US and UK) were more similar and found lower normative data in developing regions (e.g. China and Taiwan). Most of these studies stratified data into age and sex subgroups and found higher HGS in males as compared to females at all ages and a gradual decline with increasing age. Likewise, HGS continues to decline after stratifying the data by sex, dominant and non-dominant hand, and right and left hand, as age increases [15].

The focus of recent research has been on the correlates of HGS which were documented in various studies. Auyeung et al. [2] studied the sex differences in the annualized HGS decline rate and found that females had a faster rate of grip strength decline compared to males, whereas other studies found faster decline rate in males than females [7, 10]. Other sociodemographic correlates such as ethnicity and occupation [6, 13]; and anthropometric correlates such as height [16, 17], upper arm circumference [18], and waist circumference [19] were also found to be associated with HGS. Other than correlates, recent studies also shown significant positive associations of HGS with physical and mental health, such as dementia among older adults in Singapore [20], cardiovascular mortality, and stroke [21].

Singapore is a Southeast Asian country with a population of 5.54 million of which 3.90 million comprise the resident population. The multi-ethnic population has a majority of those belonging to the Chinese ethnicity (74.3%), followed by Malays (13.3%), Indians (9.1%), and others (3.2%) [22]. The average life expectancy has increased over the years. For males it is currently 80.6 years (2004: 77.1 years) while for females it is 85.1 years (2004: 82.0 years). As of 2015, there were a total of 700,208 older adults aged 60 years or above, accounting for 17.9% of the total Singapore population [22].

Malhotra et al. [6] recently published normative values for HGS using data obtained from the national Social Isolation, Health, and Lifestyles Survey (SIHLS), conducted in 2009. However, this study only assessed the sociodemographic correlates of HGS such as age, sex, ethnicity, education level and occupation, but did not account for anthropometric correlates of HGS such as, upper arm and waist circumference. Both correlate with HGS [18, 19]. Furthermore no comparisons were made between data from Singapore with that of Western and other Asian countries to understand the HGS performance of older adults in Singapore.

The current study aimed to: (1) establish the normative HGS values in the Singaporean older adults stratified by age, sex, and ethnicity; (2) compare Singapore older adults’ HGS data to Western and other Asian countries; (3) examine sociodemographic correlates of HGS, and; (4) explore the relationship of HGS with other anthropometric measurements (i.e. height, weight, upper arm circumference, and waist circumference) controlling for sociodemographic correlates.

## Methods

### Study participants

Study approval was obtained from the relevant ethics committees in Singapore -National Healthcare Group Domain Specific Review Board and the SingHealth Centralised Institutional Review Board. Older adults aged 60 years and above were randomly selected from a national database which consisted of administrative data (i.e. name, ethnicity, gender, and residential address) of all citizens and permanent residents in Singapore. These selected participants were notified of the study by mail and then approached at home by interviewing staff. Older adults who resided in day care centres, nursing homes, and institutions were also included. Individuals who were not living in Singapore and who could not be contacted due to invalid addresses were excluded from the study. Written informed consent was obtained from all the participants. In the case where participants were unable to provide consent, consent was taken from their legally acceptable representative or next-of-kin. For each older adult, an informant of each participant- “someone who knew the participant best”, was also invited to take part in the survey. The informant c﻿ould be either caregivers, co-residents or someone who had close contact with the older person but was not involved in a caregiver role. Informants p﻿rovided information on participants only when the participant could not provide the relevant information, such as questions on “participant background information” and “physical health condition” [23]. Disproportionate stratified sampling design was used where residents in the older age group and those from Malay and Indian ethnic group were over-sampled to ensure that sufficient sample size would be achieved to improve the reliability of estimates for the subgroups analysis. A detailed description of the methodology can be found in an earlier paper [23].

### Materials and assessments

All measurements and data on sociodemographic information were collected by trained interviewers. Hand dominance was determined by asking which hand they use for writing or which hand they would predominantly use when performing a task. HGS was measured in kilograms (kg) by taking the average of the two dominant handgrip attempts using a Jamar Plus + Digital Hand Dynamometer (Pennsylvania, United States). The device measures the isometric muscle contractions as the participant grip against the resistance of the stationary grip handle. For each of the HGS assessments, subjects were instructed by trained interviewers to sit as per the American Society of Hand Therapist’s (ASHT) recommendation for HGS; with their shoulder adducted and neutrally rotated, elbow flexed at 90^o^ with the forearm in neutral position for HGS measurement [24].

Sociodemographic information on age, gender, ethnicity, marital status, education, employment status were collected from participants and verified with informants. Anthropometric measurements were carried out with a brief fully structured physical and neurological assessment, the NEUROEX [25], which included height, weight, HGS, upper arm circumference, and waist circumference. Upper arm circumference was measured in centimetres using a measuring tape around the thickest part of the upper arm of the dominant hand [26]. Participants were instructed to have their shoulders relaxed and both arms hanging loosely at the sides as flexing or tightening the arm muscle would results in an inaccurate measurement. Waist circumference measured in centimetres was measured at the narrowest part of the body between the chest and hips for females, and measured at the level of the umbilicus for males [27].

### Statistical analysis

Data analysis was carried out using Statistical Analysis Software (SAS) system version (9.3). To ensure that the survey findings were representative of the Singapore population, all estimates were analysed using survey weights to adjust for complex survey data. We have computed survey weights that incorporate sampling weight, non-response weight and post-stratification weight which was used to weigh the sample back to the population to adjust for oversampling, non-response and post-stratify by age and ethnicity distributions between the survey sample and the Singapore elderly population. This approach has been recommended when analysing complex survey data [28]. Those diagnosed with dementia using 10/66 diagnostic criteria [29] were excluded from the analysis since dementia is associated with poor HGS [20, 30].

Mean and standard deviation were calculated for continuous variables, and frequencies and percentages were calculated for categorical variables. HGS were calculated and presented separately by age, sex and ethnicity. We used 6 age groups categories to present the data: 60–64 years, 65–69 years, 70–74 years, 75–79 years, 80–84 years and 85+ years. Multiple linear regression analyses were used to explore the sociodemographic and anthropometric correlates of HGS. Standard errors (SE) of means, regression coefficients and other statistics were estimated using the Taylor series’ linearization method to adjust for the weighting. Multivariate significance was evaluated using Wald X^2^ tests based on design corrected coefficient variance-covariance matrices. Statistical significance was evaluated at the 0.05 level using 2-sided tests.

## Results

### Characteristics of study participants

The descriptive data are listed in Table 1. A total of 2565 older residents participated in the study, giving a response rate of 66%. Of the 2565 participants who completed the study, 2043 participants aged 60 years and over were included in our study sample. Those with missing HGS data (*n* = 171, mostly because of health reasons) and dementia (*n* = 399) were excluded. Further, the results for those who were left-handed were excluded due to small sample size (*n* = 84). The mean age was 68.8 years, ranging from 60 to 105 years. 82.9% were of Chinese descent, 9.3% were Malays descent, 6.2% were of Indian descent, and 1.7% belonged to other ethnic groups. Majority of the sample were women (54.3%), married/ cohabiting (66.8%), had completed primary education (25.1%), and were employed (37.5%).

**Table 1:** Sociodemographic profile of sample

	Sample (mean = 22.25) (*N* = 2043)				
	Unweighted n	Weighted %	SE	Weighted Mean	SE
Age group (years)					
60–64	616	33.3	1.44	24.68	0.53
65–69	477	28.0	1.40	23.28	0.50
70–74	277	19.0	1.27	20.72	0.56
75–79	325	11.0	0.50	18.66	0.50
80–84	177	5.9	0.50	18.78	0.74
85+	171	2.8	0.00	14.94	0.60
Gender					
Men	949	45.7	1.55	28.27	0.36
Women	1094	54.3	1.55	17.18	0.22
Ethnicity					
Chinese	792	82.9	0.03	22.41	0.31
Malay	574	9.3	0.01	20.92	0.42
Indian	641	6.2	0.03	21.60	0.34
Others	36	1.7	0.01	24.41	1.83
Marital status					
Never married	114	8.2	0.91	22.47	0.97
Married/cohabiting	1299	66.8	1.45	23.49	0.33
Widowed	530	18.9	1.11	17.51	0.41
Divorced/separated	100	6.2	0.78	23.06	1.31
Education					
None	307	13.1	1.00	18.34	0.64
Some, but did not complete primary	489	23.8	1.32	21.07	0.50
Completed primary	542	25.1	1.35	23.11	0.58
Completed secondary	467	24.3	1.35	23.10	0.52
Completed tertiary	235	13.8	1.11	25.10	0.74
Employment status					
Paid work (full-time and part-time)	652	37.5	1.50	25.47	0.46
Unemployed	30	1.5	0.39	27.13	2.23
Homemaker	592	24.5	1.32	16.30	0.30
Retired	752	36.4	1.47	22.67	0.43

### Hand-grip strength by age, sex, and ethnicity

Table 2 shows the means and standard deviation of HGS by age group, sex and ethnicity. The mean HGS for the males and females participants in the youngest age group (60-64years) was 31.1 kg and 18.2 kg respectively while it dropped to 18.5 kg for males and 12.4 kg for females participants in the oldest age group (85+ ﻿years﻿). The mean HGS showed a decreasing trend with increasing age among all three ethnic groups in both sexes.

**Table 2:** Means and standard deviations (SD) of hand-grip strength (kg) by age, sex and ethnicity groups

	Females					Males				
		Total	Chinese	Malay	Indian		Total	Chinese	Malay	Indian
Age group (years)	n	Mean (SD)	Mean (SD)	Mean (SD)	Mean (SD)	n	Mean (SD)	Mean (SD)	Mean (SD)	Mean (SD)
60–64	322	18.17(5.16)	18.58(4.78)	15.83(4.14)	18.01(4.82)	294	31.14(7.85)	31.24(7.63)	30.36(9.23)	28.97(6.83)
65–69	261	18.61(4.20)	18.93(3.98)	15.85(4.14)	17.06(4.58)	216	29.31(7.15)	29.80(7.24)	27.27(6.32)	27.37(6.76)
70–74	153	16.39﻿(﻿4.22)	16.61(4.61)	14.66(4.31)	16.26(4.90)	124	26.34(5.39)	26.36(5.44)	24.80(6.18)	24.07(6.33)
75–79	189	14.74(5.85)	15.29(5.65)	12.08(4.72)	13.38(4.59)	136	23.68(6.60)	23.80(6.16)	24.27(10.10)	22.57(6.76)
80–84	86	13.82(4.80)	14.12(4.74)	10.75(4.96)	12.68(4.78)	91	24.16(7.81)	24.27(6.34)	24.80(9.92)	23.77(7.79)
85+	83	12.36(5.47)	12.79(6.47)	10.34(6.60)	11.24(5.57)	88	18.46(13.77)	18.12(13.21)	18.97(12.47)	18.14(12.18)

### Sociodemographic correlates of hand-grip strength

Table 3 shows the sociodemographic correlates of HGS. HGS was significantly greater in the youngest age group (60-64) than the other older age groups, 70-74 (β = −3.29, *p* < 0.001), 75-79 (β = −5.28, *p* < 0.001), 80-84 (β = −5.94, *p* < 0.001), and 85+ (β = −9.15, *p* < 0.001) and in males (β =10.76, *p* < 0.001) than females. Those of Malay (β = −2.01, *p* < 0.001) and Indian (β = −1.64, *p* < 0.001) ethnicity had significantly lower HGS values than Chinese after adjusting for other sociodemographic correlates.

**Table 3:** Sociodemographic correlates of hand-grip strength

	Beta coefficient	95% CI	*p* value
Age group (years)			
60–64	Reference		
65–69	−0.71	−1.78, 0.35	0.190
70–74	−3.29	−4.49, −2.10	**<.0001**
75–79	−5.28	−6.50, −4.05	**<.0001**
80–84	−5.94	−7.40, −4.47	**<.0001**
85+	−9.15	−10.78, −7.51	**<.0001**
Gender			
Men	10.76	9.79, 11.73	**<.0001**
Women	Reference		
Ethnicity			
Chinese	Reference		
Malay	−2.01	−2.82, −1.20	**<.0001**
Indian	−1.64	−2.36, −0.91	**<.0001**
Others	1.80	−0.98, 4.58	0.204
Marital status			
Never married	Reference		
Married/cohabiting	0.28	−1.30, 1.87	0.725
Widowed	0.66	−1.04, 2.36	0.445
Divorced/separated	0.46	−1.94, 2.87	0.706
Education			
None	0.65	−0.95, 2.25	0.425
Some, but did not complete primary	−0.84	−2.11, 0.42	0.192
Completed primary	−0.32	−1.64, 0.99	0.629
Completed secondary	−0.77	−2.05, 0.51	0.239
Completed tertiary	Reference		
Employment status			
Paid work (full-time and part-time)	Reference		
Unemployed	0.47	−3.29, 4.22	0.808
Homemaker	−0.98	−2.05, 0.09	0.072
Retired	−0.14	−1.18, 0.91	0.795

*CI* = confidence interval ﻿﻿

Note: Bolded values are statistically significant (*p* ≤ .0001)﻿﻿

### Multivariate analysis to test the association of anthropometric measurements with hand-grip strength

Table 4 shows the association between HGS with height, weight, upper arm circumference, and waist circumference after adjusting for sociodemographic and other anthropometric correlates. Multiple linear regression were conducted and for the overall sample, HGS was found to be significantly associated with height (β = 0.12, *p* = 0.001), weight (β = 0.09, *p* = 0.019), and inversely associated with waist circumference (β = −0.08, *p* = 0.018).

**Table 4:** Association between hand-grip strength, height, weight, upper arm circumference, and waist circumference

	Beta coefficient	95% CI	*p* value
Overall*			
Height	0.12	0.05, 0.19	**0.001**
Weight	0.09	0.02, 0.17	**0.019**
Upper arm circumference	0.11	−0.02, 0.24	0.107
Waist circumference	−0.08	−0.15, −0.01	**0.018**
Males*			
Height	0.17	0.04, 0.30	**0.010**
Weight	0.05	−0.11, 0.20	0.567
Upper arm circumference	0.33	0.11, 0.56	**0.004**
Waist circumference	−0.09	−0.23, 0.06	0.247
Females*			
Height	0.10	0.02, 0.18	**0.016**
Weight	0.13	0.03, 0.22	**0.009**
Upper arm circumference	0.03	−0.16, 0.22	0.761
Waist circumference	−0.09	−0.16, −0.01	**0.020**

*CI* = confidence interval

*Adjusted for sociodemographic correlates and other anthropometric measurements

N﻿o﻿te: Bo﻿lded values are statistically significant (*p* ≤ .05)

Stratified regression analysis showed that for males, only height (β = 0.17, *p =* 0.010) and upper a﻿rm circumference (β = 0.33, *p =* 0.004) were significantly positively associated with HGS. For females, the findings were similar to the overall sample where HGS was significantly associated with height (β = 0.10, *p =* 0.016), weight (β = 0.13, *p =* 0.009), and waist circumference (β = −0.09, *p =* 0.020).

### Comparison between Singapore and other countries

**Fig. 1:**
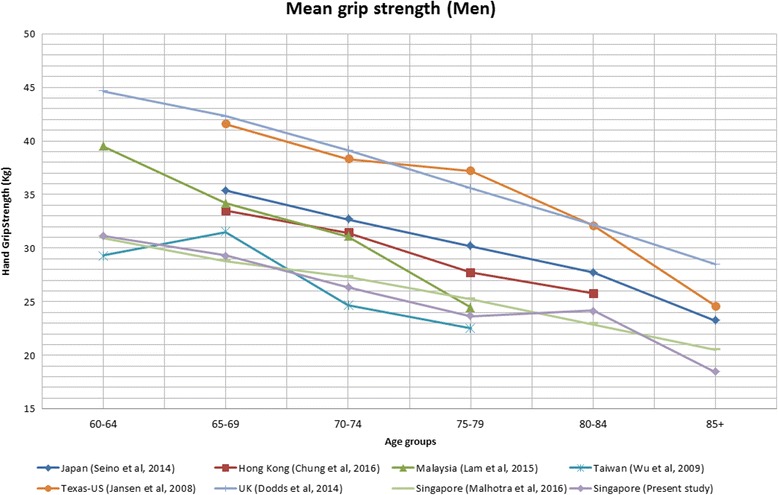
Mean grip strength of older men over six successive age ranges from 60 years old

**Fig. 2:**
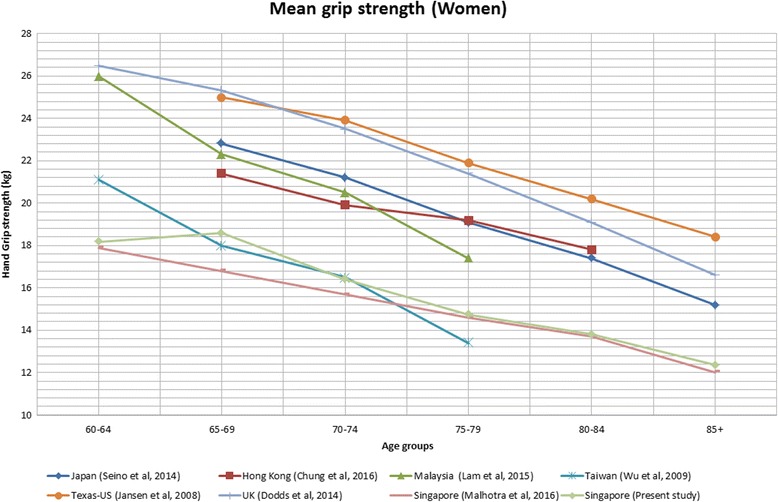
Mean grip strength of older women over six successive age ranges from 60 years old

The summary details of the eight included studies for HGS comparison are listed in Table 5. HGS data was compared with Western (i.e. US, UK) and other Asian countries (i.e. Japan, Taiwan, Hong Kong, Malaysia and Singapore). For both sexes, HGS among Singapore older adults were relatively low compared to Western and other Asian countries except for Taiwan (Figs. 1 and 2). US and UK had the highest mean HGS followed by Japan, Malaysia, Hong Kong, Singapore, and Taiwan. Only right-handed HGS data were extracted from other studies for comparison.

**Table 5:** Summary of 8 studies contributing to the comparison of hand-grip strength among countries

Study (population)	Location	N	Age range	Year(s) of data collection	Ways of Measuring	Position	Repetitions/hands/value used
Present study (WiSE study)	Singapore	2043	60-105	2012 - 2013	Jamar digital	Seated	Two/dominant(right)/mean
SIHLS* [6]	Singapore	2664	60-89	2009	Smedley spring	Standing	Two/both/mean
From 12 population studies [8]	United Kingdom	49,964	Varies	Ranges, 1990 - 2012	Jamar (*n* = 7)Smedley (*n* = 2)Nottingham electronic (*n* = 2)Takei (*n* = 1)	Seated (*n* = 8)Standing (*n* = 4)	Six/both/max (*n* = 10) Four/both/max (2)
From health fairs, geriatric primary-care clinic, and senior-citizen community events [7]	United States	224	65-92	Not specified	Jamar hydraulic	Seated	Three/both/mean
From community and hospitals [13]	Malaysia	362	≥ 60	Not specified	Jamar hydraulic	Seated	Three/both/mean
TMIG-LISA 6 cohort studies [9]	Japan	4683	≥ 65	Ranges, 1992 - 2011	Smedley	Standing	Two/dominant/max (*n* = 3) One/dominant (*n* = 3)
Convenience sample [11]	Taiwan	482	20-80+	Not specified	Jamar	Seated	Two/both(values from right hand used in analyses)/mean
Community sample [10]	Hong Kong	944	65- 84	2014 - 2015	Not specified	Not specified	Three/not specified/max

*WiSE* = Well-b﻿eing of the Singapore Elderly, *SIHLS ﻿=﻿ * Social Isolation, Health, and Lifestyles Survey, *TMIG-LISA =﻿* Tokyo Metropolitan Institute of Gerontology Longitudinal Interdisciplinary Study on Aging

## Discussion

In this article we examined the HGS values among the Singaporean older adults, as well as the sociodemographic correlates and its association with anthropometric correlates. Our results showed significant association between HGS and sociodemographic correlates; age, sex, and ethnicity. Significant associations were also found between HGS and anthropometric measurement; height, weight, upper arm circumference and waist circumference. Particular attention was paid to the sex differences in the association of HGS with anthropometric measurements. In females, increasing height, weight and decreasing waist circumference were associated with HGS, while in males, only increasing height and upper arm circumference were associated with HGS.

Our findings are in good agreement with other studies which found an association between HGS with other sociodemographic factors such as age, sex, and ethnicity [13, 31, 32]. Possible underlying mechanisms have been proposed for the association between HGS with age, sex, and ethnicity. As individuals' age, their bodies would experience age-related degenerative changes in the musculoskeletal, vascular, and nervous systems. These degenerative changes would cause deterioration of hand function in older adults and affect the hand structure such as joints, muscle, tendon, bone, nerve and receptors, blood supply, skin, and fingernails [33]. Furthermore, studies have reported major reduction of muscle mass and ability to activate the biceps brachii muscle as one ages [34]. For sex differences, Miller et al. [35] compared body mass, muscles fibres number, fibres size and fibres area between young males and females. Compared to females, males were stronger relative to lean body mass and had significantly larger type 1 fibre areas and mean fibre areas in biceps brachii. Males were reported to have a larger number of muscle fibres which contribute to the greater strength than females [35].

Few studies have looked into the ethnic differences in grip strength among populations in Southeast Asia [13, 36]. In a study conducted in rural Pahang, Malaysia, ethnic differences in grip strength was reported where the aborigines had significantly lower grip strength compared to the Malaysian Malays, Chinese, and Indians [36]. Genetic variation [37], health status and different lifestyle could account for the observed differences between Chinese, Malays, and Indians [38].

Consistent with other studies, significant associations were found between HGS with height, weight, and waist circumference in older adults [19, 39, 40]. For height, Samaras et al. [41] indicated that taller people have greater absolute strength. Absolute strength is related to muscle cross-sectional area and is correlated with the body surface area or the square of body height. Other than the cross-sectional area, factors such as nutrition in early life are reported to have positive influence on individuals’ grip strength [42]. Larger waist circumference, which is a clinical indicator of central obesity, is associated with lower grip strength [19]. Abdominal fat secretes cytokines and hormones (adipokines) and a relationship between higher cytokines levels and lower muscle mass and lower muscle strength has been reported [43]. Negative relationship between adipokines and strength and aerobic fitness in older adults has also been reported [44].

Our paper presents an interesting view of gender differences in the association of HGS with other anthropometric measurements. Compared to the overall and females’ data, for males there was a significant association between HGS and upper arm circumference but not with weight and waist circumference. Possible reasons could be due to the employment-type differences between men and women. According to the Labour Force Survey in Singapore 2015 [45], the resident employment participation rate for older men aged 65 and over was higher than older women aged 65 and over (36.0% vs. 17.6%). Among employed residents aged 60 and over, more men engaged in occupations i.e. “Production & Transport Operators, Cleaners & Labourers” (78.0% vs. 43.3%) than women, while more women engaged in “Clerical, Sales & Service Workers” (37.6% vs. 31.5%) than men. In the present study, there were more males than females in skilled labourer, 21% vs. 5%. Given that labour-intensive jobs require workers to have good physical condition and strength, it is plausible that men who engaged in these jobs have greater upper arm strength and therefore a significant association of upper arm circumference with HGS. Further research on the role of gender on the relationship between different anthropometric correlates (i.e. upper arm circumference and waist circumference) with HGS is necessary to extend our knowledge of HGS further.

### Comparison with other countries

Overall, Singapore older adults’ mean HGS was lower compared to other countries. The HGS for both genders were generally lower compared to older adults in UK [8], US [7], Japan [9], Hong Kong [10] and Malaysia [13] but was comparable to a previous study conducted in Singapore [6] and Taiwan [13]. Several possible reasons could explain the difference in normative HGS data between countries and these include differences in body composition such as mean height, weight, body sizes, palm size and ways of measuring grip strength e.g. sitting or standing positions and the brand of dynamometer [11].

The norms of HGS may differ between populations within Europe and East Asia. The Survey of Health, Ageing and Retirement in Europe (SHARE) study conducted in 11 European countries found lower HGS in the southern countries (Spain, Italy, and Greece) compared to northern and continental countries (Sweden, Denmark, Netherlands, Germany, Austria, Switzerland, and France) [46]. In a study by Lin et al. [47] which assessed the anthropometric characteristics of adults from East Asian countries (i.e. China, Taiwan, Japan, and South Korea), significant morphological difference were reported among these peoples in the same region [47]. Clearly these differences in anthropometric measurements within regions are likely to be explained by a range of factors such as nutrition and genetic factors which may also account for the differences seen in HGS among countries [48].

Varying methods of measuring grip strength could also explain the difference in grip strength across and within countries. The previous study mentioned- SHARE study - which included data from 11 European countries, used the hand-grip dynamometer, Smedley, while another systematic review using data from 12 British general population studies different dynamometers (Smedley and Jamar) in the seated and standing position (refer to Table 5) were used for the data collection. A systematic review by Roberts et al. [49] found a wide variability in the choice of grip strength measuring equipments and protocols across clinical and epidemiological studies. Furthermore, evidence pointed that variation in approach can affect the values recorded and summary measures of grip strength varied widely including maximum or mean value, from one, two or three attempts, with either hand or the dominant hand alone [49].

Within Singapore, the present study reported similar normative HGS data as Malhotra et al. [6], yet the minimal differences in data reported could be attributed to possible reasons such as individual differences, cohort effect (national survey conducted in 2009 vs. present study: 2013), and instrument used to measure HGS (Smedley spring-type dynamometer vs. present study: Jamar digital-type dynamometer), and position (standing position vs. present study: sitting position). All these findings highlight the importance of having a standardized method of assessing HGS to facilitate comparison between studies and enable consistent measurement of grip strength [49, 50].

### Limitations and strengths

The findings of this study should be interpreted in the light of the following limitations. Firstly, the generalizability of the study is limited. The present paper only includes participants with HGS measurements, not diagnosed with 10/66 dementia, and right-handed individuals. Future studies could explore the anthropometric correlates of HGS for both right and left hands. Secondly, there is also the possibility that the participants, who are older, may not have understood the instructions during HGS measurement and that could affect the validity of the measurements taken [20, 51]. To reduce the likelihood of such occurrence, all interviewers received standardized instructions and training from senior researchers to ensure proper use of the equipment and demonstrations of using the Jamar dynamometer were conducted for all subjects. All participants were also briefed in the language chosen by participants according to their familiarity and comfort.

Despite these limitations stated above, results from this study hold important implications on the healthcare of the older adults in Singapore. In terms of external comparisons, Singapore older adults’ have generally lower HGS compared to other countries. The comparison allows clinicians to gauge the performance of Singapore older adults’ HGS performance and offer a better standard for treatment and interventions, and researchers could use the normative data as baseline to study the trend for comparison with future studies. For internal comparisons, the normative grip strength data allow individuals to interpret what is typical in their country. It served as a reference point for comparison to someone of the same age, gender, and ethnicity to determine if their personal HGS is higher or lower than what is typical in their country. However, care must be taken for comparison. Since height, weight, upper arm circumference, and waist circumference were found to be associated with HGS, thus the result would be more useful as a gauge than a strict benchmark [11].

Our study was the first in Singapore that examines the anthropometric correlates of HGS in Singapore older adults and makes comparison with the available HGS data of Western and other Asian countries. Our results suggest that ethnicity and gender differences exist for the anthropometric correlates of HGS (males: height and upper arm circumference; females: height, weight, and waist circumference) in Singapore, which might also explain some of the differences between Singapore older adults against other countries.

## Conclusions

The present study demonstrated that sociodemographic correlates (i.e. age, sex, and ethnicity) and anthropometric correlates (i.e. height, weight, upper arm circumference and waist circumference) were associated with HGS in Singapore older adults. Moreover, the study found that Singaporean older adults had weaker grip strength than that of older adults from Western and other Asian countries.

## Abbreviations

ASHT: American Society of Hand Therapist
HGS: Hand-grip strength
SHARE: Survey of Health, Ageing and Retirement in Europe
SIHLS: Social Isolation, Health, and Lifestyles Survey
WiSE: Well-being of Singapore elderly
